# Cortical beta oscillations are associated with motor performance following visuomotor learning

**DOI:** 10.1016/j.neuroimage.2019.03.079

**Published:** 2019-07-15

**Authors:** Svenja Espenhahn, Bernadette C.M. van Wijk, Holly E. Rossiter, Archy O. de Berker, Nell D. Redman, Jane Rondina, Joern Diedrichsen, Nick S. Ward

**Affiliations:** a Department of Clinical and Movement Neurosciences, Institute of Neurology, University College London, 33 Queen Square, WC1N 3BG, London, UK; b Department of Radiology, Cumming School of Medicine, University of Calgary, 2500, University Drive NW, Calgary, AB T2N 4N1, Canada; c Integrative Model-based Cognitive Neuroscience Research Unit, Department of Psychology, University of Amsterdam, Postbus 15926, 1001, NK, Amsterdam, the Netherlands; d Wellcome Centre for Human Neuroimaging, Institute of Neurology, University College London, 12 Queen Square, WC1N 3BG, London, UK; e Cardiff University Brain Research Imaging Centre, School of Psychology, Cardiff University, Maindy Road, CF24 4HQ, Cardiff, UK; f Brain and Mind Institute, University of Western Ontario, London, ON N6A 3K7, Canada

**Keywords:** Ageing, Beta oscillations, EEG, Motor learning, Plasticity, Sensorimotor cortex

## Abstract

People vary in their capacity to learn and retain new motor skills. Although the relationship between neuronal oscillations in the beta frequency range (15–30 Hz) and motor behaviour is well established, the electrophysiological mechanisms underlying individual differences in motor learning are incompletely understood. Here, we investigated the degree to which measures of resting and movement-related beta power from sensorimotor cortex account for inter-individual differences in motor learning behaviour in the young and elderly. Twenty young (18–30 years) and twenty elderly (62–77 years) healthy adults were trained on a novel wrist flexion/extension tracking task and subsequently retested at two different time points (45–60 min and 24 h after initial training). Scalp EEG was recorded during a separate simple motor task before each training and retest session.

Although short-term motor learning was comparable between young and elderly individuals, there was considerable variability within groups with subsequent analysis aiming to find the predictors of this variability. As expected, performance during the training phase was the best predictor of performance at later time points. However, regression analysis revealed that movement-related beta activity significantly explained additional variance in individual performance levels 45–60 min, but not 24 h after initial training. In the context of disease, these findings suggest that measurements of beta-band activity may offer novel targets for therapeutic interventions designed to promote rehabilitative outcomes.

## 1 Introduction

The ability to learn and retain new motor skills is pivotal for everyday motor activities and sustained independence in senior adults (Seidler et al., 2010). As the old adage goes “practice makes perfect”, motor skills initially improve with training. Motor skills also continue to develop after practice has ended through a process of memory consolidation (Halsband and Lange, 2006; Robertson et al., 2004). However, people show considerable inter-individual heterogeneity in their capacity to learn, which may be of clinical significance in the context of brain pathology such as stroke (Stinear, 2010). Understanding the neurophysiological processes underlying between-subject variability in skill acquisition and consolidation may offer novel therapeutic targets for promoting long-term rehabilitative outcomes after brain injury (Stinear, 2010; Ward, 2017).

Imaging studies have revealed considerable experience-dependent plasticity of sensorimotor cortex representations during motor skill acquisition (Halsband and Lange, 2006; Karni et al., 1995; Muellbacher et al., 2002; Nudo et al., 1996; Robertson et al., 2005; Sanes and Donoghue, 2000). Learning requires plasticity and although plasticity does not necessarily lead to learning, differences in the potential for plasticity might explain variability in learning. One candidate biomarker for the potential for plasticity is the balance between GABAergic inhibitory and glutamatergic excitatory processes in the brain (Bavelier et al., 2010; Benali et al., 2008), which is reflected in the amplitude of oscillations as detected by electroencephalography (EEG) (Jensen et al., 2005; Murakami and Okada, 2006; Traub et al., 2004; Yamawaki et al., 2008). Here, we are interested in motor system plasticity, and sensorimotor cortex oscillations in the beta (15–30 Hz) frequency range are fundamental for motor control (Engel and Fries, 2010; Joundi et al., 2012; Pogosyan et al., 2009). It is well established that beta-band oscillations are dominant at rest, are suppressed during movement (Movement-Related Beta Desynchronization, MRBD) and show a rebound after movement cessation (Post-Movement Beta Rebound, PMBR) (Pfurtscheller et al., 1998; Pfurtscheller and Lopes Da Silva, 1999; Salmelin and Hari, 1994; Stancak and Pfurtscheller, 1995). Despite the upsurge in the interest in neuronal oscillations and in particular beta-band oscillations due to their potential role as markers of motor system function and dysfunction (Nicolo et al., 2015; Takemi et al., 2015; Ward, 2015; Wu et al., 2015), the extent to which cortical oscillations in the beta frequency relate to individual differences in motor learning behaviour remains incompletely understood.

Here, we explored the neurophysiological mechanisms associated with individual differences in short-term motor learning behaviour using EEG. We included both young and elderly subjects in order to maximise inter-subject variability, because (i) alterations in beta oscillations have been seen with ageing (Gaetz et al., 2010; Heinrichs-Graham and Wilson, 2016; Rossiter et al., 2014), and (ii) previous studies have suggested an age-related reduction in the potential for plasticity (Chollet, 2013; Fathi et al., 2010; Tecchio et al., 2008; Todd et al., 2010). Specifically, given the link between beta oscillations and both inhibitory GABAergic activity (Hall et al., 2011, 2010; Jensen et al., 2005; Muthukumaraswamy et al., 2013) and learning (Boonstra et al., 2007; Houweling et al., 2008; Pollok et al., 2014), we assessed the extent to which beta oscillatory power can explain differences in motor learning behaviour. Specifically, we explored whether the pre- and/or post-training state of cortical activity is of functional relevance for short-term motor learning.

## 2 Methods

### 2.1 Subjects

Twenty young (range 18–30 years, 1 left-handed; for more details see Table 1) and twenty elderly (range 62–77 years, 1 left-handed) subjects took part in our study over two consecutive days. Two subjects were excluded because they either did not comply with the task requirements or later disclosed a neurological disease. All included subjects (N = 38) had normal or corrected-to-normal vision and fulfilled the following inclusion criteria: (a) no history of neurological or psychiatric disease; (b) no physical disability of the arms or wrists; (c) no use of drugs affecting the central nervous system or self-reported abuse of any drugs; and (d) age within specified range (18–30 years or 60–80 years). To minimize circadian fluctuations in beta oscillatory levels (Toth et al., 2007; Wilson et al., 2014), all subjects were tested in the time between 9am and 2pm. In addition, subjects were instructed to abstain from alcohol and caffeine the evening and morning before the testing. The study was approved by the National Hospital for Neurology and Neurosurgery, UCL Hospitals NHS Foundation Trust and the local research ethics committee at University College London where the study was conducted. All subjects gave written informed consent in accordance with the Declaration of Helsinki.

At the beginning of the experiment, subjects underwent assessments of upper limb motor ability (Nine Hole Peg Test, NHPT; grip strength using dynamometer) and cognitive functioning (Sustained Attention to Response Test, SART). Since sleep has been shown to affect motor memory consolidation (Korman et al., 2007; Walker et al., 2002; Wilson et al., 2012), on both days, subjects additionally provided information about their sleep quantity and quality (computerised version of St. Mary's Hospital sleep questionnaire adapted from (Ellis et al., 1981)) for the nights preceding testing.

### 2.2 Experimental design

**Fig. 1:**
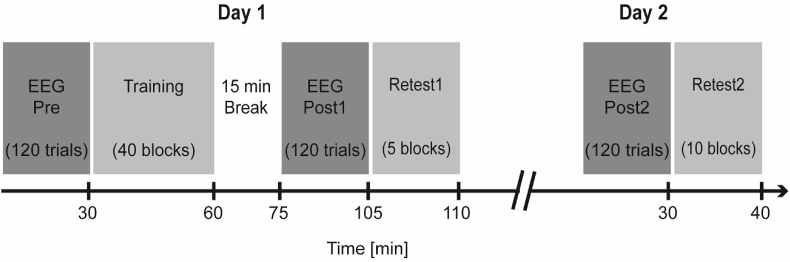
Timeline of experiment. EEG was recorded during the performance of a simple wrist flexion/extension task before (Pre) and at two time points after the training phase (Post1 and Post2). Performance on the motor learning task was retested after a time delay on the same day (retest1 on day 1, 45–60 min after initial training) and the following day (retest2 on day 2, 24h after initial training).

**Fig. 2:**
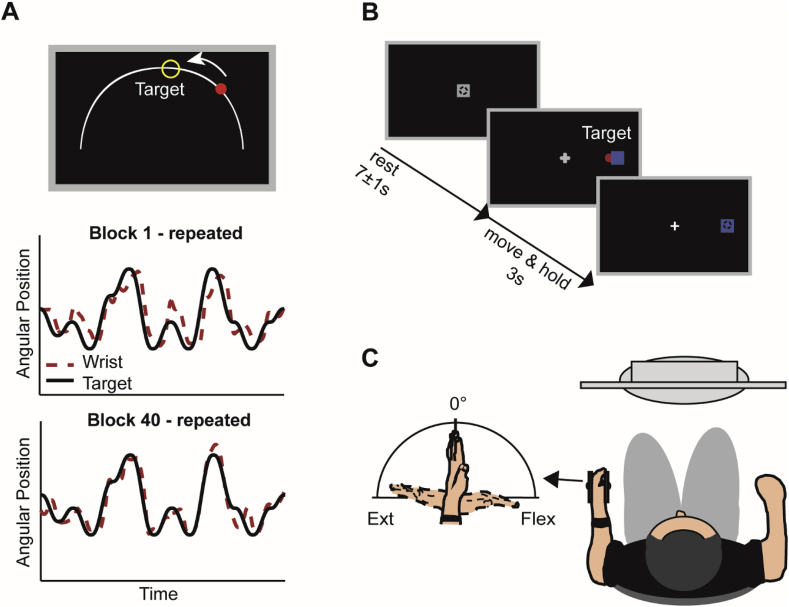
*Experimental setup and paradigms.****A****, Subjects were trained to track a target (yellow circle) moving back and forth along a fixed arc as accurately and smoothly as possible. Online visual feedback in terms of a colour change of the wrist cursor (red to green) was provided at times when the wrist cursor was located inside the circular target. Original recordings during the continuous tracking task at the beginning and end of the initial training are shown for the repeated sequence of an example participant (A, lower panel). The solid black line represents the motion of the target, while the dashed red line represents the motion of the wrist.****B,****For the simple wrist flexion/extension task, subjects were instructed to perform wrist flexion and extension to move the wrist cursor (red circle) from the initial start position (grey square) to one of two target positions (blue square) upon target presentation.****C****, During both tasks, subjects sat in front of a computer monitor with their non-dominant hand rested in a wrist rig that restricted movement to flexion and extension around the wrist joint.*

The experimental design is illustrated in Fig. 1. All subjects trained with the wrist of their non-dominant arm on a continuous tracking task over a single training session (40 blocks; 20–40 min) with the aim of improving motor performance beyond pre-training levels. The tracking task involved two types of sequences within each block, a random and a repeated sequence (see below). Improvement on the random sequence is a measure of general skill learning, whilst any additional improvement on the repeated sequence reflects sequence-specific motor learning of the precise sequence pattern (Wulf and Schmidt, 1997). Motor performance was defined as the accuracy with which subject's wrist movement tracked the target movement (Fig. 2A). Participants' motor performance was retested at two different time points: 45–60 min (retest1 on day 1; 5 blocks) and 24 h (retest2 on day 2; 10 blocks) after initial training. These retest sessions allowed (i) temporary effects (e.g. fatigue or boredom) that build up over the course of training (Brawn et al., 2010; Rickard et al., 2008) to dissipate, thus only leaving the fairly stable learning effects and (ii) consolidation of motor memories to occur, which may result in retention, decrement or even enhancement of the previously acquired motor skill after a night's sleep (Robertson et al., 2004; Walker, 2005).

Electroencephalography (EEG) recorded during the performance of a simple wrist flexion/extension task was used to assess changes in pre-movement (resting) and movement-related beta activity before (Pre), immediately after (Post1) and 24h after (Post2) the initial training phase. By recording beta oscillatory activity during the performance of a separate task, not used for training, but which employed comparable motion features (flexion and extension), it was possible to investigate the generic properties of brain activity and their relation to motor learning. The simple wrist flexion/extension task is known to induce clear movement-related changes in beta activity that are distinct and separate in time (Espenhahn et al., 2016). This is not the case for continuous movements where these beta dynamics start to overlap until they are no longer clearly distinguishable with increasing movement tempo (Houweling et al., 2010). Here, we were interested in linking well-established features of movement-related beta dynamics (MRBD and PMBR) separately to motor learning.

### 2.3 Apparatus and tasks

All tasks were performed with the non-dominant hand resting in an instrumented wrist rig (modified from (Turk et al., 2008)). The rig restricted movement to flexion and extension around the wrist joint in the horizontal plane and ensured minimal hand and arm movement during the experiment. Wrist angular displacement was sensed by a built-in potentiometer, with a displacement of 0° indicating a neutral position of the wrist, with the hand being in the same plane as the forearm. The angular position of the wrist, sampled at 100Hz, was continuously displayed on a computer monitor as a cursor in the form of a red circle – hereafter referred to as “wrist cursor”. The target was displayed either as an open yellow circle (continuous tracking task) or as a blue square (simple motor task). On day 1, prior to the motor tasks, the mid-point and maxima of an individual's maximum active range of movement (AROM) around the wrist joint was measured and subsequently used as start and/or target positions in the continuous tracking task and simple motor task, respectively. Stimuli were presented using custom software routines written in Matlab (version R2013b; The MathWorks, Inc., Natick, MA, USA).

#### 2.3.1 Continuous tracking task

Subjects were required to continuously track a circular target (in yellow) that moved back and forth along a fixed arc through a predefined sequence of 12 positions (Fig. 2A). The minimum jerk approach (Flash and Hogan, 1985; Hogan, 1984) was employed to ensure smooth target motion through the sequence positions. The maximum range of the target trajectory was defined as ±45° of wrist flexion and extension, and the target always started and finished at the individual mid-point position of each subject's AROM.

Each block consisted of two sequences, one random and one repeated sequence presented in randomised order, with a 3s stationary target between both. The repeated sequence was identical throughout initial training (40 blocks), and retest sessions (retest1 on day 1: 5 blocks; retest2 on day 2: 10 blocks) and randomly selected from a pool of 57 difficulty-matched sequences. Each random sequence was encountered only once; however, the same set of difficulty-matched sequences was used across subjects. Subjects were instructed to move their wrist so as to shift the red wrist cursor to match the movement of the target as ‘accurately and smoothly as possible’.

Prior to the training, the average velocity with which the target moved along the arc was individually determined in order to ensure that the task was of equal difficulty for everyone at the beginning of the training and left enough room for improvement in performance. For this purpose, we implemented an adaptive staircase procedure, which, on any given trial, adjusted (increased/decreased) the target velocity dependent on the subject's preceding performance until a pre-specified criterion range was reached. On average, subjects reached the criterion in 14.4 ± 4.5 trials and there was no difference in the number of trials required between groups (*t*_*(1,36)*_ = 0.94, *p* = 0.072). The individually determined target velocity with which subjects were subsequently trained on the continuous tracking task was applied to all sessions and did not significantly differ between young (mean velocity ±SD = 55.38 ± 6.92 deg/s) and elderly subjects (mean velocity ±SD = 50.78 ± 9.41 deg/s) [*t*_(36)_ = 1.71, *p* = 0.095].

During initial training and retest sessions, online visual feedback in terms of a colour change of the wrist cursor (from red to green) was provided at times when the subject positioned the wrist cursor inside the circular target. In addition, at the end of each block, subjects were made aware of their change in motor performance by presenting a score on the screen. Prior to the start of training, subjects received explicit verbal information regarding the presence of a repeated sequence along with a random sequence in every block. However, they were not shown the repeated sequence. To determine the time point at which participants gained explicit knowledge of the repeated sequence, after each block they had to decide (forced-choice) which of the two sequences within each block the repeated sequence was - i.e. tell the experimenter whether it was the first or second sequence they tracked within the block. The trajectories of the target and subject's wrist cursor did not leave a residual trail on the screen and hence, subjects could not visualize the entire target sequence.

#### 2.3.2 Simple wrist flexion and extension task

The EEG measures we used as explanatory variables of motor learning were acquired separately from the learning task using a simple visually-cued wrist flexion and extension task (Espenhahn et al., 2016). During each trial, wrist movements were always initiated from the same start position displayed at the centre of the screen that represented the mid-point of a subject's individual AROM. The cue to perform wrist flexion or extension movements was the random appearance of one of two targets (in blue), on the left or right, equidistant from the central start position (Fig. 2B). Each of the targets represented the subject's maximum wrist flexion or extension position. Subjects were instructed to move their wrist upon presentation of the target so as to shift the red wrist cursor from the central start position to match the position of the target in a ‘quick and discrete’ movement. The target position was displayed for 3s and subjects had to maintain the wrist cursor inside the target until being cued to return to the initial start position. Once subjects returned to the start position, the next cue to move was delivered following a delay of 7±1s. The task comprised 120 trials, and subjects were instructed to minimize eye movements by focusing on a centrally located fixation cross. Movement onset was defined as the time when the angular velocity of the wrist exceeded a threshold of 5% of the maximum velocity and sustained this speed for at least 100 ​ms. Movement termination was defined as the time when the velocity fell below the threshold for that trial for at least 500 ​ms. For each subject, we discarded trials in which the movement was initiated before the cue signal, reaction time was excessively long (>mean ​+ ​2.5 SD), or movement time was excessively long/short (>/< mean ​± ​2.5 SD) (average ∼8% of trials). This resulted on average in 110 ​± ​4 remaining trials. Reaction time (RT, interval between visual cue and movement onset), movement time (MT, interval between movement onset and movement termination), and peak velocity (PV) were calculated and averaged per experimental condition. Since movement time and peak velocity were highly correlated (*r* > 0.8), we report only reaction time and movement time.

### 2.4 EEG recording

Scalp EEG (ANT Neuro, Asalab, The Netherlands) was continuously recorded at 2084Hz using 64 electrodes mounted on an elastic cap (waveguard EEG cap) according to the international 10–20 EEG system. The impedance was kept below ≤5kΩ and the EEG signal was referenced to Cz during recording. The timing of the visual cue (blue target) in the motor task was marked in the simultaneous EEG recording, with separate markers for each condition (flexion, extension). Surface electromyography (EMG) using bipolar electrodes in a belly-tendon montage placed on the wrist extensor (extensor carpi radialis longus) and flexor (flexor carpi radialis) muscles monitored movements of the non-dominant hand.

### 2.5 Data analysis

#### 2.5.1 Motor learning

Motor performance on the continuous tracking task, was parametrized by Root Mean Square Error (RMSE), an established measure implemented by other motor learning studies (Al-Sharman and Siengsukon, 2014; Boyd and Winstein, 2006; Roig et al., 2014; Siengsukon and Boyd, 2009). RMSE captures the deviation of the wrist position at time *i* (*w*_i_) from the target position (*t*_*i*_), and serves as a composite measure of temporal and spatial measurements of time lag and distance as calculated using the following equation:

$$\text{RMSE}=\sqrt{{\overset{N}{\underset{i=1}{\sum }}\left(t_{i}-w_{i}\right)}^{2}/N}$$

where *N* is the total number of time samples of the sequence in each block. Thereby, smaller RMSE values reflect better motor performance.

**Fig. 3:**
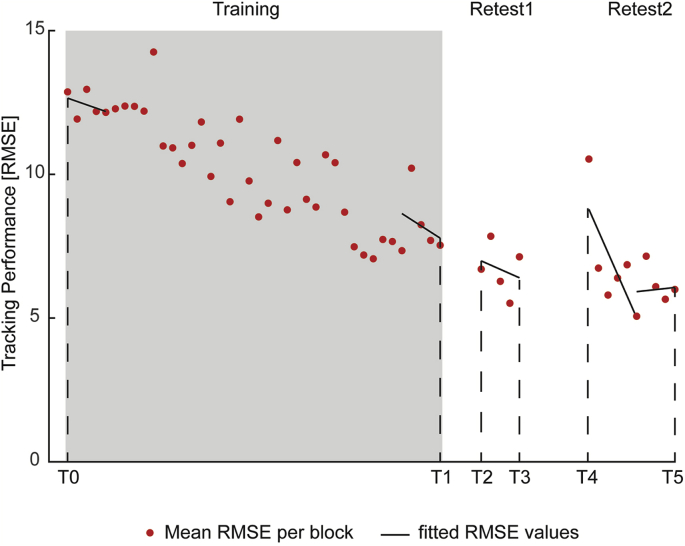
Linear regression approach for exemplary subject. Dots represent individual blocks of an example subject during training and retest sessions of repeated sequence only. Black lines represent linear regression models across 5 blocks at beginning and end of individual sessions. Corrected performance estimates were derived from these linear regression models at six different time points (T0 = first block of training, T1 = last block of training, T2 = first block of retest1, T3 = last block of retest1, T4 = first block of retest2, and T5 = last block of retest2) and used to subsequently assess changes in performance with training.

RMSE was calculated for repeated and random sequences separately and averaged across each block of the training and retest sessions. As the beginning and end of individual training and retest sessions might not be representative of actual motor performance (e.g. due to warm-up decrement at the beginning or fatigue at the end) (Adams, 1961), a linear regression model was fitted across the first and last 5 blocks of individual training and retest sessions (approach adopted from (Waters-Metenier et al., 2014)). This fit provided a corrected performance estimate of the first and last blocks of each session (Fig. 3). Please note that performance refers to this corrected performance estimate unless stated otherwise.

The analysis then concentrated on six time points in order to assess changes in motor performance across time: first block of training (T0), last block of training (T1), first block of retest1 (T2), last block of retest1 (T3), first block of retest2 (T4), and last block of retest2 (T5). As outlined above, various processes can occur during time periods in which the task is not practised (i.e. between T1 and T2 or T3 and T4), such as dissipation of temporary effects (e.g. fatigue or boredom) (Brawn et al., 2010; Rickard et al., 2008) and motor memory consolidation (Hotermans et al., 2006; Robertson et al., 2004; Walker, 2005). As such, motor performance at T2 is most likely to reflect fairly stable learning effects unaffected by training-induced temporary effects such as fatigue or boredom, while performance at T4 likely indexes retention of the acquired motor skill overnight, due to motor memory consolidation.

#### 2.5.2 Spectral power

Pre-processing and time-frequency analysis of EEG data during the performance of the simple motor task were performed using SPM12 (Wellcome Centre for Human Neuroimaging, http://fil.ion.ucl.ac.uk/spm) and additional scripts written in Matlab (version R2016a; The MathWorks Inc., Natick, MA, USA). The raw EEG signal was first offline re-referenced to the average signal across all electrodes, bandpass filtered between 5 and 100Hz, additionally filtered with a 50Hz notch filter to reduce line noise contamination, and downsampled to 300Hz. Data were epoched from −1 to 9s relative to visual cue onset (0s). Poorly performed trials (see section 2.3.2) were excluded and the remaining EEG trials were visually scrutinized. Trials containing artefacts (e.g. muscle activation or large eye blinks) were additionally removed. For each session, on average 91 ± 12 and 87 ± 15 artefact-free EEG trials remained for younger and older subjects, respectively, and the number of trials did not differ between conditions (*p* > 0.1) or groups (*p* > 0.3, repeated-measures ANOVA). Artefact-free EEG time-series from each single trial were decomposed into their time-frequency representations in the 5–45Hz range with frequency steps of 0.1Hz. A 7-cycle Morlet wavelet was used for the continuous wavelet transformation. Power (*P*) was averaged across trials and rescaled in order to show changes relative to the corresponding pre-movement baseline period (-1–0s prior to cue onset), expressed as percentage of this baseline power (*P*_*ref*_):

$$\text{\%}\;\text{power}=\frac{P-P_{ref}}{P_{ref}}*100$$

Spectral power time-series were derived from a pre-selection of electrodes overlying the sensorimotor cortices, both contralateral and ipsilateral to the moving hand (MRBD: ‘C4’ ‘CP4’ ‘CP2’ and ‘C3’ ‘CP3’ ‘CP1’ for contra- and ipsilateral hemispheres, respectively; PMBR: ‘C2’ ‘C4’ ‘CP4’ and ‘C1’ ‘C3’ ‘CP3’ for contra- and ipsilateral hemispheres, respectively). These electrodes were selected based on prior findings showing that the most prominent task-related changes in beta activity were observed in these electrodes when performing the exact same simple motor task (Espenhahn et al., 2016). These bilateral electrodes were pooled as contralateral and ipsilateral regions of interest, respectively, and combined within hemispheres (‘C4’ ‘CP4’ CP2′ C2′ and ‘C3’ ‘CP3’ ‘CP1’ ‘C1’ for contra- and ipsilateral hemispheres, respectively) to derive resting beta power.

**Fig. 4:**
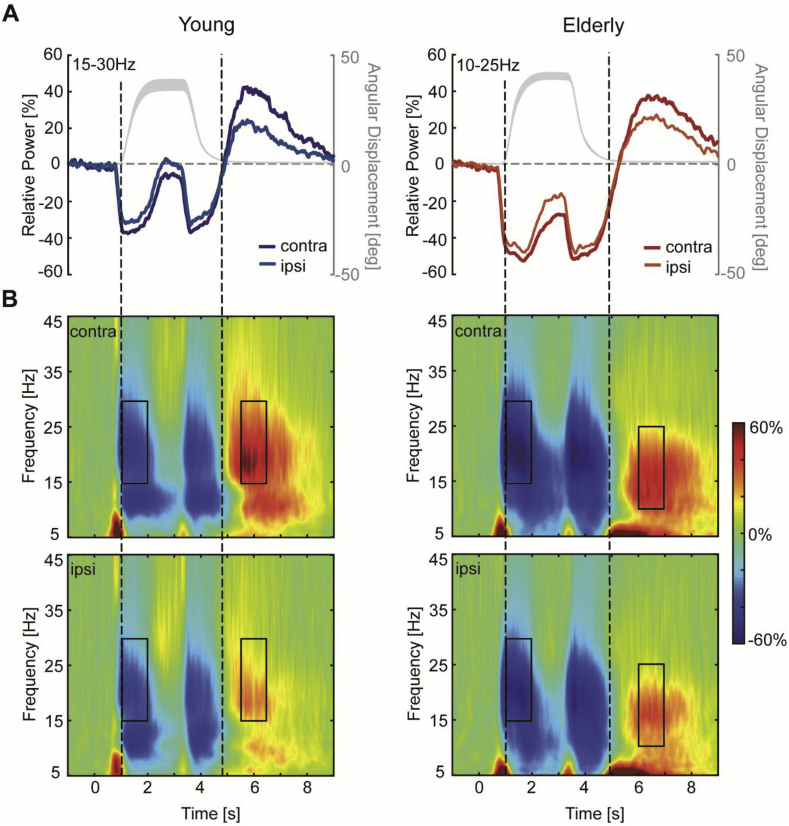
Angular displacement profile and movement-related changes in beta activity. **A**, Group-averaged angular position trajectory (grey curve) and beta power time courses for contra- and ipsilateral sensorimotor cortex for young (left panel) and elderly (right panel) subjects, respectively. Movement kinematics were similar between both groups and illustrate the movement towards the target, the static contraction/holding phase and the return movement to the initial start position. **B**, Time-frequency maps from contralateral and ipsilateral sensorimotor cortex show two distinct time windows of peak changes in beta activity (MRBD and PMBR) indicated by black rectangles. Please note that the PMBR in elderly subjects occurred at lower beta frequencies (10–25Hz) and ∼500 ms later compared to younger subjects. These time-frequency windows were tested for significant differences between groups and EEG sessions.

To select time-frequency windows of interest that were orthogonal to potential differences between conditions (flexion versus extension) when the simple motor task was performed (Pre, Post1, Post2), we averaged over conditions, sessions, and subjects for each group separately. We then chose specific time-frequency windows based on peak changes in beta activity in time-frequency maps of the bilateral sensorimotor regions, which revealed clear movement-related beta-band (15–30Hz) activity in two distinct time windows of interest. This information was used to optimize the alignment of constant duration (1s) and width (15Hz) time-frequency windows to capture maximum MRBD (1–2s relative to cue onset), occurring between cue onset and movement termination, and PMBR (young group: 5.5–6.5s relative to cue onset; elderly group: 6–7s relative to cue onset), which emerges after movement cessation. This was done for young and elderly subjects separately because of known age-related reduction of beta peak frequency (Rossiter et al., 2014). Indeed, in elderly subjects peak changes in beta activity after movement cessation appeared at lower beta frequencies (10–25Hz) and ∼500 ms later compared to younger subjects, however this could not be explained by age-related differences in return movement kinematics (Fig. 4A). Selected time-frequency windows and electrodes applied to all subjects and sessions, and were not adjusted individually.

Subsequently, for each individual subject, percentage decrease (MRBD) and increase (PMBR) in beta power were extracted from the respective 1s time windows and averaged for each EEG session (Pre, Post1 and Post2) for the pre-selected electrodes over each hemisphere. The absolute pre-movement (resting) baseline beta (BB) power from −1 to 0s relative to cue onset was also obtained and assessed for age-related differences and training-related changes.

In total, 6 different beta parameter estimates were used for subsequent analyses: pre-movement baseline beta (absolute power), MRBD (relative power) and PMBR (relative power) from contra- and ipsilateral sensorimotor cortices, respectively. Importantly, these EEG measures of resting and movement-related beta-band power have previously been shown to have high intra-subject reliability (Espenhahn et al., 2016), a prerequisite for exploring the relationship between individual neurophysiological differences and motor learning behaviour.

### 2.6 Statistical analysis

To assess how motor tracking performance changed over time, we performed a repeated-measures ANOVA, with ‘group’ (2 levels: young vs elderly) as between-subject factor and ‘sequence type’ (2 levels: repeated vs random) and ‘time’ (5 levels: T0 vs T1 vs T2 vs T3 vs T4) as within-subject factors. Additionally, to ensure comparable baseline performance and thus, allow for direct comparison between age groups, a repeated-measures ANOVA of motor performance at T0 (baseline) was employed.

Since beta oscillations have been shown to be altered with ageing (Gaetz et al., 2010; Heinrichs-Graham et al., 2018; Rossiter et al., 2014) and motor learning (Boonstra et al., 2007; Gehringer et al., 2018; Houweling et al., 2008; Mary et al., 2015; Pollok et al., 2014), measures of resting and movement-related beta activity were evaluated applying separate repeated-measures ANOVAs with ‘group’ (2 levels: young vs elderly) as between-subject factor and ‘hemisphere’ (2 levels: contralateral vs ipsilateral) and EEG ‘session’ (3 levels: Pre vs Post1 vs Post2) as within-subject factors.

A Greenhouse-Geiger correction was applied whenever Mauchly's test indicated a lack of sphericity. *Post hoc* Bonferroni-adjusted t-tests were performed whenever main effects and interaction effects were detected in the ANOVAs. Prior to ANOVAs and *post hoc* t-tests, Kolmogorov-Smirnov test was used to affirm normal distribution of the data. Results were considered significant if p-values were below 0.05. All data presented in the text and tables are represented as mean ± SD unless stated otherwise. Statistical analyses were performed using SPSS (version 22; IBM) and custom-written Matlab routines.

#### 2.6.1 Regression analysis

Finally, a multiple linear regression approach was employed in order to investigate whether spectral power measures of beta-band activity relate to individual differences in the extent of motor learning, accounting for multicollinearity between neurophysiological (Heinrichs-Graham and Wilson, 2016) and motor performance measures (see light green and blue boxes of Supplementary Fig. 1). Specifically, separate stepwise multiple linear regression models (with forward and backward algorithm; inclusion/exclusion probability levels: αEnter<0.05/αExclude>0.1) were used to select those variables that provided a unique contribution to explaining motor performance at T2 and T4 for the repeated and random sequence, respectively. Motor performance at T2 reflects fairly stable learning effects unaffected by training-induced temporary effects such as fatigue or boredom, while performance at T4 indexes retention of the acquired motor skill overnight, reflecting motor memory consolidation. Specifically, a combination of spectral power measures, including (a) baseline beta power, (b) MRBD, and (c) PMBR from both sensorimotor cortices, as well as motor performance measures during the training session, i.e. (d) at T0 and (e) at T1, were used to explain performance at T2, while motor performance measures during retest1, i.e. (f) at T2 and (g) T3, were further included to explain performance at T4. In addition, demographic information such as age, motor function, cognitive function and sleep characteristics were equally included (total number of regressors: 22 and 29 for T2 and T4, respectively). All variables were z-scored before analysis to produce regression coefficients (β) of comparable magnitude.

Significant correlations raise the potential for beta power measures to serve as predictors of learning outcome. To avoid overfitting and evaluate the predictive strength of each regression model, a leave-one-out cross-validation (LOOCV) approach was employed (Arlot and Celisse, 2010; Picard and Cook, 1984). This cross-validation method is an established procedure for assessing generalization of results to an independent data set, particularly with smaller sample sizes (Huang et al., 2011; Kang et al., 2014). The strength of the prediction model was quantified in terms of the correlation coefficient between actual and predicted motor performance. A permutation-test (100 iterations) was used to assess whether the difference between the actual and predicted performance was greater than would be expected by chance (p-value below 0.05).

## 3 Results

As expected, young and elderly subjects differed in aspects of upper limb motor ability and cognitive function (Table 1). In addition, elderly subjects reported sleeping fewer hours compared to their younger counterparts, in line with studies demonstrating a decrease in total sleep time with age (for review see (Ohayon et al., 2004)).

**Table 1:** Group characteristics of young and elderly subjects.

	Young	Elderly	Between-group difference
N	19	19	–
Age	25 ± 4	69 ± 4	***t(36) = -34.8, p < 0.001***
Male: Female ratio	8:11	7:12	*Χ*^*2*^*=0.11, p=0.740*
Handedness (Edinburgh)	94 ± 8	84 ± 21	*t(23.01)=1.86, p=0.076*
Grip Strength [lb]	34 ± 11.30	27 ± 8.33	***t(36) = 2.05, p = 0.048***
Dexterity [pegs/s]	0.67 ± 0.08	0.60 ± 0.08	***t(36) = 2.73, p = 0.010***
Sustained attention (Error score, 0–225)	8 ± 3.79	13 ± 10.70	***t(22.44) = -2.14, p = 0.043***
Sustained attention (RT in ms)	363 ± 70.11	446 ± 144.64	***t(26.02) = -2.25, p = 0.033***
Sleep Quantity [hours]^#^	7 ± 0.70	6 ± 0.96	***U = 70.0, p = 0.001***
Sleep Quality (1–8)^#^	5.6 ± 1.12	5.2 ± 0.87	*U=130.5, p=0.138*

Between-group comparisons revealed a significant difference in NHPT, grip strength, SART, and sleep quantity the previous night. For continuous data, independent-samples t-tests were used to test for between-group differences. For discrete data (^#^), Mann-Whitney U-tests were applied. Handedness was assessed using the Edinburgh Handedness Inventory (Oldfield, 1971). Upper limb functional measures are non-dominant hand only and sleep measures are averaged across both days (both sleep measures were not significantly different between day 1 and day 2, p > 0.05). Significant effects are indicated in bold. NHPT: Nine Hole Peg Test; SART: Sustained Attention to Response Test.

### 3.1 Presence of motor skill learning with healthy ageing

**Fig. 5:**
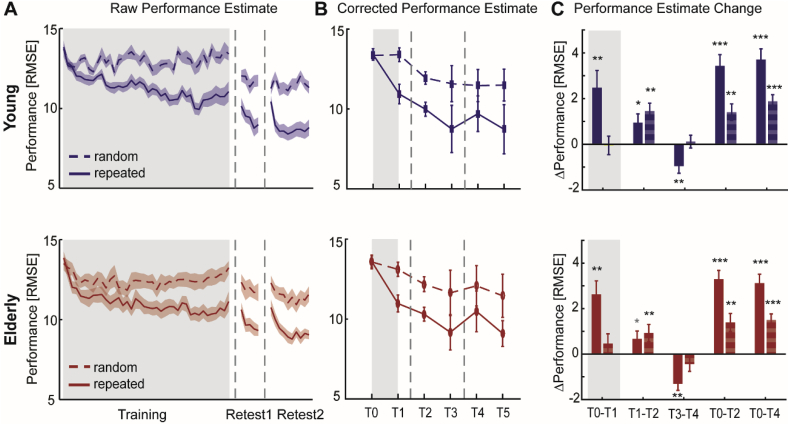
Motor skill learning of young and elderly subjects. **A**, Average motor performance (RMSE) for repeated and random sequences (solid and dashed lines respectively) across training (day 1), retest1 (day 1) and retest2 (day 2) sessions suggest comparable performance improvements of young (blue) and elderly (red) subjects. Vertical dashed lines represent breaks between each session. **B**, Corrected performance estimates at the beginning and end of training (T0, T1) and retest (retest1: T2, T3; retest2: T4, T5) sessions. **C**, Performance differences (Δ) between time points, focusing on online learning (T0-T1) and offline learning across a shorter (retest1, T1-T2) or longer (retest2, T3-T4) time delay as well as overall performance changes from baseline (T0-T2; T0-T4). Solid bars represent Δ performance on the repeated sequence and striped bars on the random sequence. Positive and negative values, respectively, signify performance improvement and decrement. Shaded area (**A**) and error bars (**B, C**) indicate between-subject SEM. Statistical difference from zero: *p ​< ​0.05, **p ​< ​0.01, ***p ​< ​0.001, grey * p ​< ​0.1 (trend).

Motor performance for both young and elderly subjects at training and retest sessions is shown in Fig. 5A. Since there were no systematic differences in baseline (block 1) performance between young and elderly groups [*F*_(1,36)_ = 0.047, *p* = 0.830] or repeated and random sequences [*F*_(1,36)_ = 0.12, *p* = 0.730], nor an interaction effect [*F*_(1,36)_ = 0.482, *p* = 0.492] (Fig. 5B), we were able to directly compare performance on the motor learning task between age groups.

A repeated-measures ANOVA on motor performance revealed a significant main effect of ‘time’ [*F*_(4,144)_ = 63.14, p < 0.001, effect size ƞ_p_^2^ = 0.637] and ‘sequence type’ [*F*_(1,36)_ = 92.56, *p* < 0.001, effect size ƞ_p_^2^ = 0.720], but no effect of age [*F*_(4,36)_ = 0.31, *p* = 0.584]. In addition, a significant ‘time x sequence type’ interaction was found [*F*_(4,144)_ = 19.74, *p* < 0.001, effect size ƞ_p_^2^ = 0.354]. *Post hoc* analyses were performed to separately assess changes in motor performance with initial training (online) and following a shorter (retest1) or longer (retest2) time delay during which subjects did not practice the task (offline).

#### 3.1.1 Performance changes over the course of training

During the training phase, motor performance improved over time (T0 vs T1) irrespective of age, but these improvements were different between repeated and random sequences and varied considerably between individuals [*F*-statistics and *p*-values of ANOVAs are summarized in Table 2]. *Post hoc* analyses revealed a significant improvement of ∼19% for the repeated sequence (Δperformance = 2.6 ± 2.9 RMSE, range = −3.9–12.3 RMSE) [*t*_*(37)*_ = 5.43, *p* < 0.001, effect size *ƞ_p_*^*2*^ = 0.443] (Fig. 5C). This was not seen for the random sequence (Δperformance = 0.2 ± 1.8 RMSE, range = −3.8–3.2 RMSE) [*t*_*(37)*_ = 0.69, *p* = 0.489], indicating that improvements in motor performance primarily occurred via a sequence-specific learning effect which appeared to be unaffected by ageing.

**Table 2:** ANOVA results of subjects’ motor performance at different time points during the motor learning process.

	Group	Time	Sequence Type	Interactions
**Performance changes across initial training**				
T0 vs T1	*F*_*(1,36)*_*=0.01, p=0.933*	***F***_***(1,36)***_*** = 17.57, p < 0.001, ƞ_p_***^***2***^*** = 0.328***	***F***_***(1,36)***_*** = 30.93, p < 0.001, ƞ_p_***^***2***^*** = 0462***	**time x sequence:** ***F***_***(1,36)***_*** = 28.33, p < 0.001, ƞ_p_***^***2***^*** = 0.440***
**Performance changes after time delay (retest1, retest2)**				
T1 vs T2	*F*_*(1,36)*_*=0.02, p=0.895*	***F***_***(1,36)***_*** = 25.97, p < 0.001, ƞ_p_***^***2***^*** = 0.419***	***F***_***(1,36)***_*** = 65.49, p < 0.001, ƞ_p_***^***2***^*** = 0.645***	n.s.
T3 vs T4	*F*_*(1,36)*_*=0.86, p=0.361*	***F***_***(1,36)***_*** = 20.81, p < 0.001, ƞ_p_***^***2***^*** = 0.366***	***F***_***(1,36)***_*** = 106.43, p < 0.001, ƞ_p_***^***2***^*** = 0.747***	**time x sequence:** ***F***_***(1,36)***_*** = 13.12, p = 0.001, ƞ_p_***^***2***^*** = 0.268***
**Overall performance changes from baseline**				
T0 vs T2	*F*_*(1,36)*_*=0.32, p=0.575*	***F***_***(1,36)***_*** = 93.08, p < 0.001, ƞ_p_***^***2***^*** = 0.721***	***F***_***(1,36)***_*** = 19.99, p < 0.001, ƞ_p_***^***2***^*** = 0.357***	**time x sequence:** ***F***_***(1,36)***_*** = 40.99, p < 0.001, ƞ_p_***^***2***^*** = 0.532***
T0 vs T4	*F*_*(1,36)*_*=1.11, p=0.299*	***F***_***(1,36)***_*** = 129.77, p < 0.001, ƞ_p_***^***2***^*** = 0.783***	***F***_***(1,36)***_*** = 18.70, p < 0.001, ƞ_p_***^***2***^*** = 0.645***	**time x sequence:** ***F***_***(1,36)***_*** = 34.87, p < 0.001, ƞ_p_***^***2***^*** = 0.492***

Significant effects are indicated in bold. T0: beginning of training session; T1: end of training session; T2: beginning of retest1; T3: end of retest1; T4: beginning of retest2. n.s.: not significant.

#### 3.1.2 Performance changes after training

After establishing that young and elderly subjects showed a comparable ability to learn, next motor performance at retest1 was examined. During the short time delay between the end of the initial training and the retest1 session (T1 vs T2), motor performance significantly improved without further training for both the repeated (7% improvement, Δperformance = 0.8 ± 1.6 RMSE, range = −2.9–5.1 RMSE) [*t*_*(37)*_ = 3.17, *p* = 0.003, effect size *ƞ_p_*^*2*^ = 0.215] and random (9% improvement, Δperformance = 1.2 ± 1.6 RMSE, range = −2.3–5.2 RMSE) [*t*_*(37)*_ = 4.71, *p* < 0.001, effect size *ƞ_p_*^*2*^ = 0.382] sequence, indicating a boost in performance early after initial training (45–60 min) (Fig. 5C). Overall, performance significantly improved from T0 to T2 not only for the repeated sequence (25% improvement, Δperformance = 3.4 ± 1.9 RMSE, range = 0.6–9.4 RMSE) [*t*_*(37)*_ = 10.91, *p* < 0.001], but also the random sequence (10% improvement, Δperformance = 1.4 ± 1.6 RMSE, range = −3.0–5.1 RMSE) [*t*_*(37)*_ = 5.31, *p* < 0.001]. Again these changes in motor performance greatly varied between individuals as indicated by the range of performance changes.

Lastly, changes in motor performance, without practice, at 24 h (retest2) after initial training were assessed. Performance significantly deteriorated from T3 to T4 irrespective of age, but dependent on the type of sequence. While motor performance on the random sequence was retained overnight (Δperformance = –0.2 ± 1.5 RMSE, range = −3.3–3.2 RMSE) [*t*_*(37)*_ = -1.21, *p* = 0.236], significant performance decrements (i.e. overnight forgetting) of ∼13% were observed for the repeated sequence (Δperformance = −0.6 ± 1.4 RMSE, range = −4.1–2.8 RMSE) [*t*_*(37)*_ = -5.79, *p* < 0.001, effect size *ƞ_p_*^*2*^ = 0.478] (Fig. 5C). Thus, while training-related improvements in general motor performance were retained for at least 24 h, overnight forgetting that was specific to the repeated sequence occurred for both young and elderly subjects. Despite these sequence-specific offline decrements, overall performance at T4 was significantly better compared to T0 for the repeated sequence (24% improvement, Δperformance = 3.9 ± 1.9 RMSE, range = 0.9–9.9 RMSE) [*t*_*(37)*_ = 10.87, *p* < 0.001] and random sequence (12% improvement, Δperformance = 1.7 ± 1.4 RMSE, range = −1.4–4.1 RMSE) [*t*_*(37)*_ = 7.87, *p* < 0.001].

### 3.2 Changes in spectral power with age and training

All subjects were able to perform the simple motor task during EEG recording and there were no significant differences in movement kinematics between age groups for either the movement towards the target [RT: *F*_(1,36)_ = 0.02, *p* = 0.896; MT: *F*_(1,36)_ = 1.14, *p* = 0.293] nor the return movement towards the initial start position [RT: *F*_(1,36)_ = 0.61, *p* = 0.441; MT: *F*_(1,36)_ = 0.58, *p* = 0.450]. Average spectral changes in contralateral and ipsilateral sensorimotor cortices in response to wrist movement are shown in Fig. 4B before (Pre) and at two time points (Post1 and Post2) after the initial training. General features of the spectral changes in beta activity induced by the simple motor task have been detailed in a previous study (Espenhahn et al., 2016). Briefly, a reduction in beta power, MRBD, was observed in both sensorimotor cortices during movement towards the target and during return movement to the initial start position. Following return movement cessation, a strong but transient increase in beta power, PMBR, with a contralateral preponderance was observed.

#### 3.2.1 Resting beta power

**Fig. 6:**
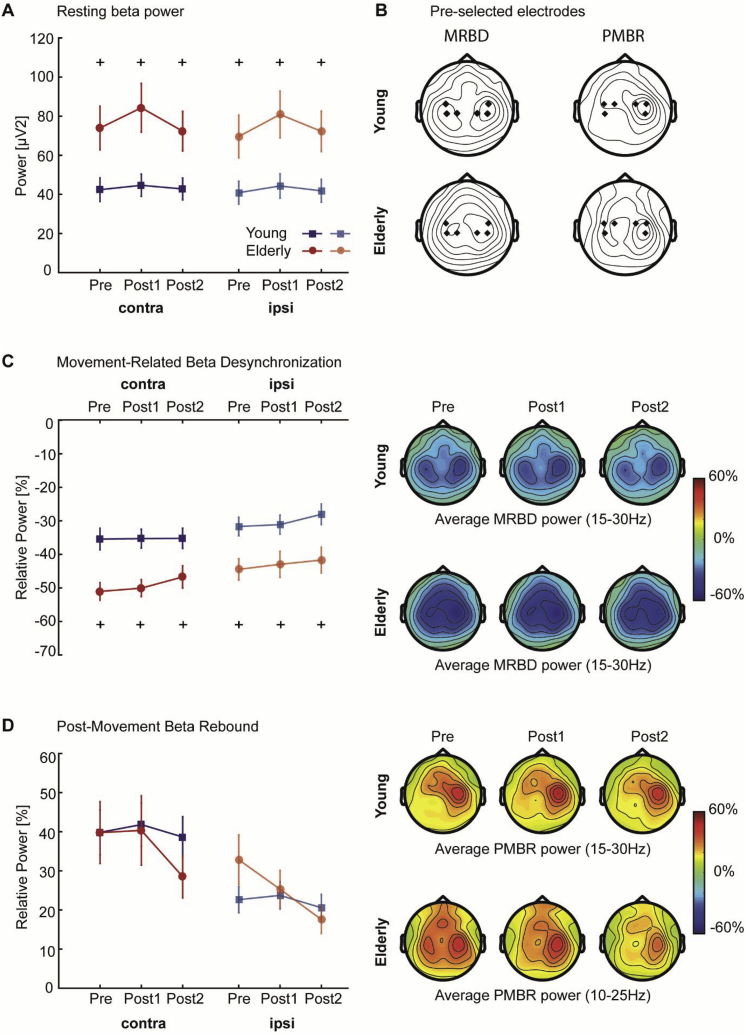
Alterations in beta power and corresponding topographic maps **A**, Average pre-movement (resting; -1–0s) beta power was significantly higher in the elderly group (red and light red) compared to the younger subjects (dark and light blue) for both sensorimotor cortices before (Pre), immediately after (Post1), and 24-h after (Post2) training. **B**, Topographical plots of grand-averaged beta power showing the pre-selected electrodes (black diamonds) which were pooled as contralateral and ipsilateral regions of interest. **C-D**, Power in the movement (1–2s; MRBD) and post-movement time window (5.5–6.5s/6–7s; PMBR) before (Pre), immediately after (Post1), and 24-h after (Post2) training derived from contralateral and ipsilateral sensorimotor cortices of young (dark and light blue) and elderly (red and light red) subjects indicated a differential effect of age upon these beta dynamics. Error bars indicate between-subject SEM. Significant between-group differences are indicated with a ‘+‘. Topographical distributions (right panels) of movement-related beta activity show differential contralateral and ipsilateral modulation patterns for MRBD and PMBR. Note, that PMBR in elderly subjects showed a bilateral distribution before training compared to the contralateral preponderance in younger subjects (**D**, right panel), but this topographical distribution shifted towards a more contralateral PMBR after the initial training.

Absolute beta power during the pre-movement (resting) baseline period was significantly affected by age, with elderly subjects exhibiting higher beta power in both contralateral and ipsilateral sensorimotor cortices (Fig. 6A, *F*-statistics and *p*-values of all ANOVAs are summarized in Table 3), consistent with previous observations (Heinrichs-Graham and Wilson, 2016; Rossiter et al., 2014). While there was no hemispheric difference in beta power and no interaction effects, absolute beta power was significantly different between sessions. *Post-hoc* analyses revealed a significant but transient increase in beta power immediately after training (Post1) in both contralateral [Pre vs Post1: *t*_(37)_ = -2.98, *p* = 0.011; Post1 vs Post2: *t*_(37)_ = 2.59, *p* = 0.032] and ipsilateral [Pre vs Post1: *t*_(37)_ = -4.60, p < 0.001; Post1 vs Post2: *t*_(37)_ = 2.48, *p* = 0.05] sensorimotor cortex which returned back to pre-training levels on day 2 [Pre vs Post2: *t*_(37)_ = 0.28, *p* = 1.00].

**Table 3:** ANOVA results for spectral power measures.

	Group	Hemisphere	Session	Interactions
BB	***F***_***(1,36)***_*** = 7.01, p = 0.012, n***_***p***_^***2***^*** = 0.163***	*F*_*(1,36)*_*=1.80, p=0.188*	***F***_***(2,72)***_*** = 7.06, p = 0.002, n***_***p***_^***2***^*** = 0.164***	*n.s.*
MRBD	***F***_***(1,36)***_*** = 10.78, p = 0.002, ƞ***_***p***_^***2***^*** = 0.230***	***F***_***(1,36)***_*** = 31.81, p < 0.001, ƞ***_***p***_^***2***^*** = 0.469***	***F***_***(2,72)***_*** = 3.29, p = 0.043, ƞ***_***p***_^***2***^**=0.084**	***3-way:*** ***F***_***(2,72)***_*** = *4.10, *p = *0.021, *ƞ***_***p***_^***2***^**=0.102**
PMBR	*F*_*(1,36)*_*=0.01, p=0.939*	***F***_***(1,36)***_*** = 21.99, p < 0.001, ƞ***_***p***_^***2***^*** = 0.379***	***F***_***(2,72)***_*** = 4.17, p = 0.019, ƞ***_***p***_^***2***^**=0.104**	*n.s.*

*Significant effects are indicated in bold. BB: Pre-*movement *baseline beta; MRBD: Movement-Related Beta Desynchronization; PMBR: Post-Movement Beta Rebound; n.s.: not significant.*

#### 3.2.2 Movement-related beta power changes

Averaged beta power changes during movement (MRBD) and after movement cessation (PMBR) in both sensorimotor cortices and topographic maps are shown in Fig. 6C–D. Interestingly, the magnitude of MRBD and PMBR was differentially affected by age. Elderly subjects showed a greater beta power decrease in both sensorimotor cortices during the movement towards the target than their younger counterparts (Fig. 6C). In contrast, the magnitude of the power increase after movement termination was not significantly different between young and elderly subjects (Fig. 6D). As expected from an unilateral task, a significant hemispheric difference in the magnitude of MRBD and PMBR indicated that both beta-band dynamics were overall more pronounced in the hemisphere contralateral to the moving hand. Also, a marginally significant effect of ‘session’ and a significant ‘group x hemisphere x session’ interaction was found for MRBD. *Post hoc* analyses indicated that the age-related difference in the magnitude of MRBD was significant in both sensorimotor cortices [contralateral sensorimotor cortex *F*_*(1,36)*_ = 12.93, *p* = 0.001, effect size *ƞ*_*p*_^*2*^ = 0.264; ipsilateral sensorimotor cortex: *F*_*(1,36)*_ = 8.12*, p* = 0.007*, ƞ*_*p*_^*2*^ = 0.184], but a significant linear reduction in the magnitude of MRBD across sessions was only found in the ipsilateral hemisphere [*F*_*(2,72)*_*=*4.26, *p* = 0.018, effect size *ƞ*_*p*_^*2*^ = 0.106].

In addition, a decrease in the magnitude of PMBR across sessions was found, but no interactions. *Post hoc* analyses showed that this decrease in PMBR across sessions was restricted to the ipsilateral sensorimotor cortex and elderly subjects only [*F*_*(2,36)*_*=*7.47, *p* = 0.002, effect size *ƞ*_*p*_^*2*^ = 0.293]. In line with this, inspection of the topographical distribution of PMBR (Fig. 6D, right panel) confirmed a training-related change in PMBR, with elderly subjects exhibiting a more bilateral distribution of PMBR prior to the training which shifted towards a contralateral preponderance following training.

### 3.3 Beta oscillations are associated with post-training motor performance

Our results so far showed that even though young and elderly subjects demonstrate comparable short-term motor learning, there are clear age-related differences in beta power measures, which might explain individual variability in motor learning performance. Thus, in order to gain insight into the role of beta activity in explaining motor learning behaviour, we employed a stepwise multiple linear regression approach within a leave-one-out cross-validation (LOOCV), including young and elderly subjects to naturally vary inter-subject differences.

**Fig. 7:**
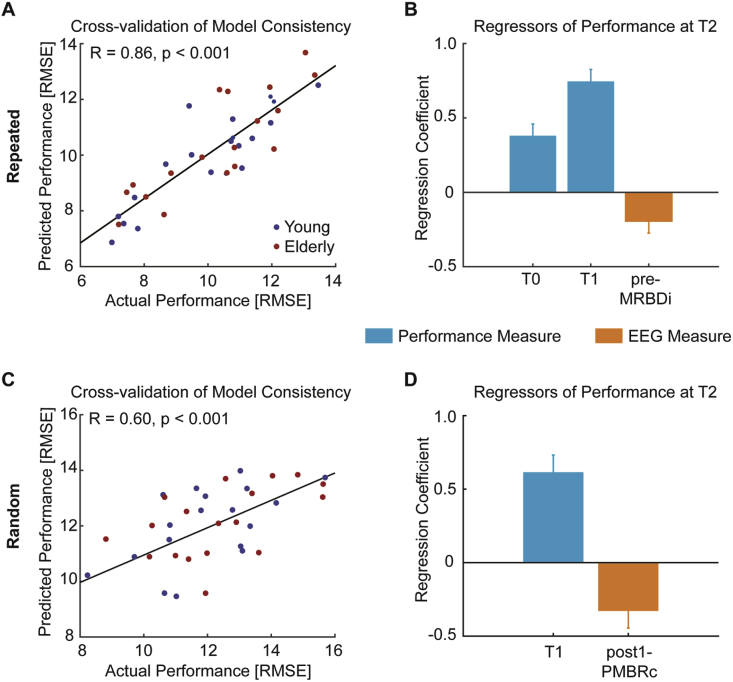
Prediction of motor performance at T2. Stepwise multiple linear regression provided statistically significant performance prediction (**A, C**) as quantified by the correlation coefficient between the actual and predicted motor performance across healthy subjects. Together, these motor performance and spectral power measures accounted for 74% and 36% of variance in performance on the repeated and random sequence, respectively. Significance of these correlations was determined by permutation-testing. **B**, Subjects' performance during training exerted the strongest effect on performance of the repeated sequence. Crucially, an additional model parameter relating to movement-related beta activity prior to training was negative, indicating that smaller magnitude of MRBD is associated with better performance. **D**, Similarly, performance on the random sequence was affected by model parameters relating to motor performance and movement-related beta activity. The negative coefficient for the beta power parameter indicates that greater magnitude of post-training PMBR is associated with better performance at T2. Z-scored regression coefficients (β) quantify the influence of each significant predictor upon performance level at T2. Error bars represent SEM.

This approach yielded models with three and two significant predictive factors that accounted for 74% (Fig. 7A) and 36% (Fig. 7C) of the variance in performance on the two types of learning (sequence-specific and general) shortly after visuomotor learning (T2), respectively. Despite performance during the training phase being the best predictor [T0: β = 0.38, *t*_*(37)*_ = 4.76, *p *< 0.001; T1: β = 0.74, *t*_*(37)*_ = 9.30, *p *< 0.001], we found that pre-training MRBD in ipsilateral sensorimotor cortex significantly accounted for performance at T2 on the repeated sequence [β = −0.19, *t*_*(37)*_* *= -2.41, *p *= 0.02] (Fig. 7B). Since the beta power decrease is expressed as a negative percentage value (relative to baseline), the negative coefficient value implies that smaller magnitude of MRBD in ipsilateral sensorimotor cortex prior to training is associated with better motor performance. Similarly, *post-hoc* pairwise correlations revealed a non-significant negative correlation between pre-training ipsilateral sensorimotor cortex MRBD and performance at T2 [r = −0.31, *p* = 0.060] (Supplementary Fig. 1), which becomes significant after regressing out performance during training as confounding covariates [partial correlation: r = −0.38, *p* = 0.021].

Further, we found that performance on the random sequence at T2 was predicted by a model incorporating not only performance during the training phase [T1: *β* = 0.62, *t*_(37)_ = 5.28, *p* < 0.001], but also post-training PMBR [*β* = −0.33*, t*_(37)_ = -2.82, *p* < 0.01] from contralateral sensorimotor cortex (Fig. 7D). The negative coefficient value for the PMBR measure implies better motor performance at T2 with greater magnitude of PMBR after training. In line, *post-hoc* pairwise correlation analysis revealed a significant negative relationship between post-training PMBR and performance at T2 on the random sequence [r = −0.40, *p* = 0.014] (Supplementary Fig. 1), with performance at the end of training influencing this relationship [partial correlation: r = −0.43, *p* = 0.008].

Finally, neither motor performance on the repeated nor random sequence 24 h after initial training (T4) was related to spectral power measures after accounting for the effect of prior motor performance. Interestingly, beyond the influence of motor performance, sleep quantity [*β* = −0.34*, t*_(37)_ = -3.70, *p* < 0.001] was associated with motor performance on the random sequence (Supplementary Fig. 2).

## 4 Discussion

In the present study, we have tested for age-related differences in visuomotor learning and in the characteristics of movement-related beta oscillations. Firstly, we found that the degree of short-term motor learning was comparable between young and elderly individuals. Secondly, we found age-related differences in beta oscillations, such that older individuals exhibited higher pre-movement baseline beta power and greater MRBD, but no differences in the magnitude of PMBR. Lastly, we found that movement-related beta oscillatory dynamics could account for different aspects of performance levels amongst all subjects shortly (45–60 min) after but not 24 h after visuomotor learning. Specifically, better performance of the repeated sequence shortly after training (T2) was related to smaller pre-training MRBD in ipsilateral motor cortex, whereas better performance of the random sequences at T2 was related to greater post-training PMBR in contralateral motor cortex, suggesting different mechanisms in learning repeated and random sequences.

Healthy ageing has been argued to reduce the ability to learn new motor skills (Boyd et al., 2008; Ehsani et al., 2015; Harrington and Haaland, 1992; Howard and Howard, 1997; McNay and Willingham, 1998; Shea et al., 2006) or exert a detrimental effect on motor memory consolidation (Brown et al., 2009; Howard and Howard, 1989; Spencer et al., 2007; Wilson et al., 2012). However, there is no consensus over the capability of the ageing brain for motor learning. Our study demonstrated preserved short-term motor learning and retention with advancing age. The absence of age-related deficits in motor learning may be attributed to the characteristics of the task employed compared to other studies (e.g. wrist vs fine finger movements) (Voelcker-Rehage, 2008) and of course may reflect age-related adaptations in motor regions and networks of the brain as described elsewhere (e.g. (Boudrias et al., 2012; Mattay et al., 2002; Reuter-Lorenz et al., 2000, 1999; Stern, 2009; Ward et al., 2008; Wu and Hallett, 2005).

While young and elderly individuals showed comparable visuomotor learning, their cortical beta oscillations during an independent motor task were significantly different. Older subjects exhibited higher pre-movement baseline beta power and greater MRBD, consistent with prior literature (Gaetz et al., 2010; Heinrichs-Graham and Wilson, 2016; Rossiter et al., 2014). Interestingly, no age-related differences in the magnitude of PMBR were observed. Taken together, the differential effect of age on MRBD and PMBR together with their well described differential modulation in contra- and ipsilateral hemispheres (Van Wijk et al., 2012) is interesting and suggests that these beta-band dynamics are maturationally distinct with distinct functional significance (Hall et al., 2011; Muthukumaraswamy et al., 2013). At a mechanistic level, a wealth of animal and human literature suggests that oscillatory activity in the beta-band reflects the balance of excitation and inhibition within reciprocally connected networks of inhibitory GABAergic interneurons and excitatory glutamatergic pyramidal cells (Gaetz et al., 2011; Hall et al., 2011, 2010; Jensen et al., 2005; Muthukumaraswamy et al., 2013; Roopun et al., 2006; Yamawaki et al., 2008). Based on previous pharmaco-MEG studies (Hall et al., 2011, 2010; Jensen et al., 2005; Muthukumaraswamy et al., 2013; Roopun et al., 2006; Yamawaki et al., 2008), the age-related changes in beta power at rest and during movement observed in our study could reflect increased GABAergic inhibition in older subjects. The fact that PMBR appears unaffected by age, suggests that its relationship with GABAergic signalling is different than that of baseline beta and MRBD. However, despite evidence for a link between cortical beta oscillations and GABAergic inhibition, we did not directly measure GABA, and thus, the inferences about GABAergic inhibition in this study are merely speculative based on measurement of beta oscillations.

A small number of studies have reported changes in beta oscillations in the sensorimotor cortex in the context of motor learning, reporting greater MRBD and PMBR after training (Boonstra et al., 2007; Houweling et al., 2008; Mary et al., 2015; Moisello et al., 2015; Nelson et al., 2017; Pollok et al., 2014). It has been argued that these changes might represent early plastic processes in this area associated with motor learning. However, it is also possible that the changes are due to the improvements in the learned behaviour itself, and so represent a performance confound. In this study, we purposely selected an independent non-learned motor task with which to probe beta oscillatory dynamics, and did not find movement-related beta activity to be enhanced following motor training. We did however find that, as in previous studies (Moisello et al., 2015; Nelson et al., 2017), pre-movement resting beta power was significantly enhanced after training. This transient training-related modulation of beta power might be related to a reduction of cortical excitability that is akin to temporary suppression of cortical plasticity with motor learning (Cantarero et al., 2013; Rioult-Pedotti et al., 2007, 2000; 1998; Rosenkranz et al., 2007; Stefan et al., 2006; Ziemann et al., 2004) as it returned to original pre-training levels after a night's sleep.

Having looked for age-related changes in learning and beta dynamics, we next wanted to understand whether variability in beta dynamics could account for variability in learning. Here, we employed a regression approach with LOOCV, to examine for variables that might predict performance at T2 and at T4. Performance at T2 and T4 were, as expected, strongly dependent on the subject's initial performance (see Supplementary Fig. 1 for correlations between variables), but our approach allowed us to ask whether different aspects of beta dynamics could account for additional varaibility in final performance over and above initial performance. Specifically, subjects who exhibited smaller MRBD prior to training performed better on the repeated sequence after training. On the other hand, greater post-training level of PMBR was identified as a significant predictor of better performance on the random sequence. Given that, as we have discussed, MRBD and PMBR are likely to have distinct mechanistic underpinnngs, our results also suggest that the two types of learning (sequence-specific and general) are dependent on different neural processes.

Smaller pre-training MRBD, likely reflecting reduced GABAergic inhibition (Hall et al., 2011, 2010; Muthukumaraswamy et al., 2013), might facilitate the induction of motor cortical plasticity and result in better motor performance. Rather unexpectedly, MRBD in the ipsilateral rather than contralateral sensorimotor cortex was related to sequence-specific motor performance at T2. Ipsilateral suppression of beta oscillatory activity during unimanual movement is a well-established phenomenon (Gross et al., 2005; Pfurtscheller et al., 1996; Salmelin and Hari, 1994), but its functional role is not fully understood. It has been proposed that ipsilateral MRBD does not merely reflect interhemispheric ‘cross-talk’ between motor cortices that facilitates movements, but may be a consequence of neural processes inhibiting mirror movements through interhemispheric inhibition (Jurkiewicz et al., 2006; Van Wijk et al., 2012). Since surface electromyography (EMG) was not recorded from both hands, it cannot be verified whether reduced ipsilateral MRBD was associated with the occurrence of mirror movements, even though subjects were instructed to relax their non-moving hand and were monitored by the experimenter. Alternatively, smaller ipsilateral MRBD also suggests that the suppression of beta power during movement is more lateralized towards the contralateral hemisphere. It could be speculated that individuals with smaller ipsilateral MRBD have slightly more dexterous unimanual motor control and therefore, perform better.

Greater post-training PMBR might reflect neural processes that facilitate practice-dependent sensorimotor reorganization after training. While beta activity, and by inference PMBR, has been suggested to promote the status quo of motor states (Engel and Fries, 2010; Gilbertson et al., 2005) and has been associated with the processing of sensory afference (Alegre et al., 2002; Cassim et al., 2001), Tan and colleagues have recently proposed a unifying theory in which PMBR is modulated by the history of task-relevant errors and is related to the uncertainty associated with feedforward predictions (Tan et al., 2016, 2014). An alternative explanation might thus be that greater post-training PMBR, reflecting better accuracy (or less error) during the previous training, might then preserve motor commands or forward models that require little updating. However, the current work was not designed to study the role of beta-band dynamics for error monitoring, and thus, this interpretation is purely speculative.

Despite beta activity being linked to motor performance on the same day as training, motor performance 24 h after training (overnight), was not associated with beta oscillatory measures. One potential explanation for this lack of relationship may be that other factors have important implications for motor skill retention. For example, sleep has been suggested to play a fundamental role in retention of motor learning, with a wide belief that it benefits motor memory consolidation (Al-Sharman and Siengsukon, 2014; Diekelmann and Born, 2010; Fischer et al., 2002; Nettersheim et al., 2015; Walker, 2005; Walker et al., 2002). Interestingly, our findings support this notion as longer sleep duration the night prior to the retest2 session appeared beneficial for retention of general motor performance (random sequence) (see Supplementary Fig. 2).

A number of limitations are worth discussing in more detail. For example, while motor sequence learning has been shown to elicit widespread activity changes in the cortical-striatal network (Dayan and Cohen, 2011; Doyon et al., 2003), the current study focused on beta oscillatory activity in sensorimotor cortex only. This was not meant to imply that training-dependent plasticity was confined to sensorimotor cortex, but rather was based on previous work demonstrating the crucial role of sensorimotor cortex for motor learning and early consolidation (Muellbacher et al., 2002; Nudo et al., 1996; Plautz et al., 2000; Robertson et al., 2005). Further, although the experimental design attempted to minimize the accumulation of fatigue during training by providing subjects with ample rest between blocks, closer inspection of motor performance in Fig. 5 still suggests a small decline in performance towards the end of the training phase. While we purposely selected an independent non-learned task with very similar motion features as the visuomotor learning task to probe beta activity, we cannot entirely rule out that the difference in the nature of the task (discrete vs continuous) might have led to a reduced effect size in our study. Lastly, the definition of motor learning in practice is not without ambiguity and hence a diversity of analytical approaches are employed in experimental studies. Inaccurate deduction of learning caused by inadequate metric selection, might for example suggest a failure of training, when in fact poor choice of outcome measures rather than a lack of efficacy of training is the problem. Importantly, rather than using normalized performance (e.g. relative to baseline) which might be conceptually fraught (Kitago and Krakauer, 2010), we assessed learning based on absolute performance levels. Currently the lack of standard procedures regarding the choice of outcome measures (Huang and Krakauer, 2009) makes comparisons between motor learning studies difficult. Clearly, further work is required to understand the complex relationship between neuronal activity and motor learning, including a unified approach to adequate motor learning metrics. Future studies should also manipulate the balance between excitatory and inhibitory mechanisms in order to evaluate the concurrent changes in beta oscillatory dynamics and motor learning behaviour.

In conclusion, the current findings imply that accessible measurements of beta activity reflect meaningful individual differences in the motor system that can be utilized in basic research and clinical studies. Movement-related beta desynchronization and post-movement beta rebound explained additional variability in individual post-learning performance differences. Given the complexity of the human nervous system, it might not be surprising that cortical oscillations may be only one of several factors important for motor learning. Notwithstanding, EEG/MEG studies of cortical dynamics in humans have the potential to bridge the gap between cellular and behavioural accounts of cortical plasticity (Ward, 2017). In the context of disease, these findings suggest that measurements of beta-band activity may offer novel targets for therapeutic interventions designed to promote rehabilitative outcomes (Ward, 2017).

## Conflicts of interest

There is no conflict of interest.
