# Supplementary material for: Cortical beta oscillations are associated with motor performance following visuomotor learning
Source: Neuroimage. 2019 Jul 15;195:340–53. doi: 10.1016/j.neuroimage.2019.03.079 (PMC6547051; doi:10.1016/j.neuroimage.2019.03.079)
Supplement: Multimedia component 1 [file mmc1.docx]

**Supplementary Figures**


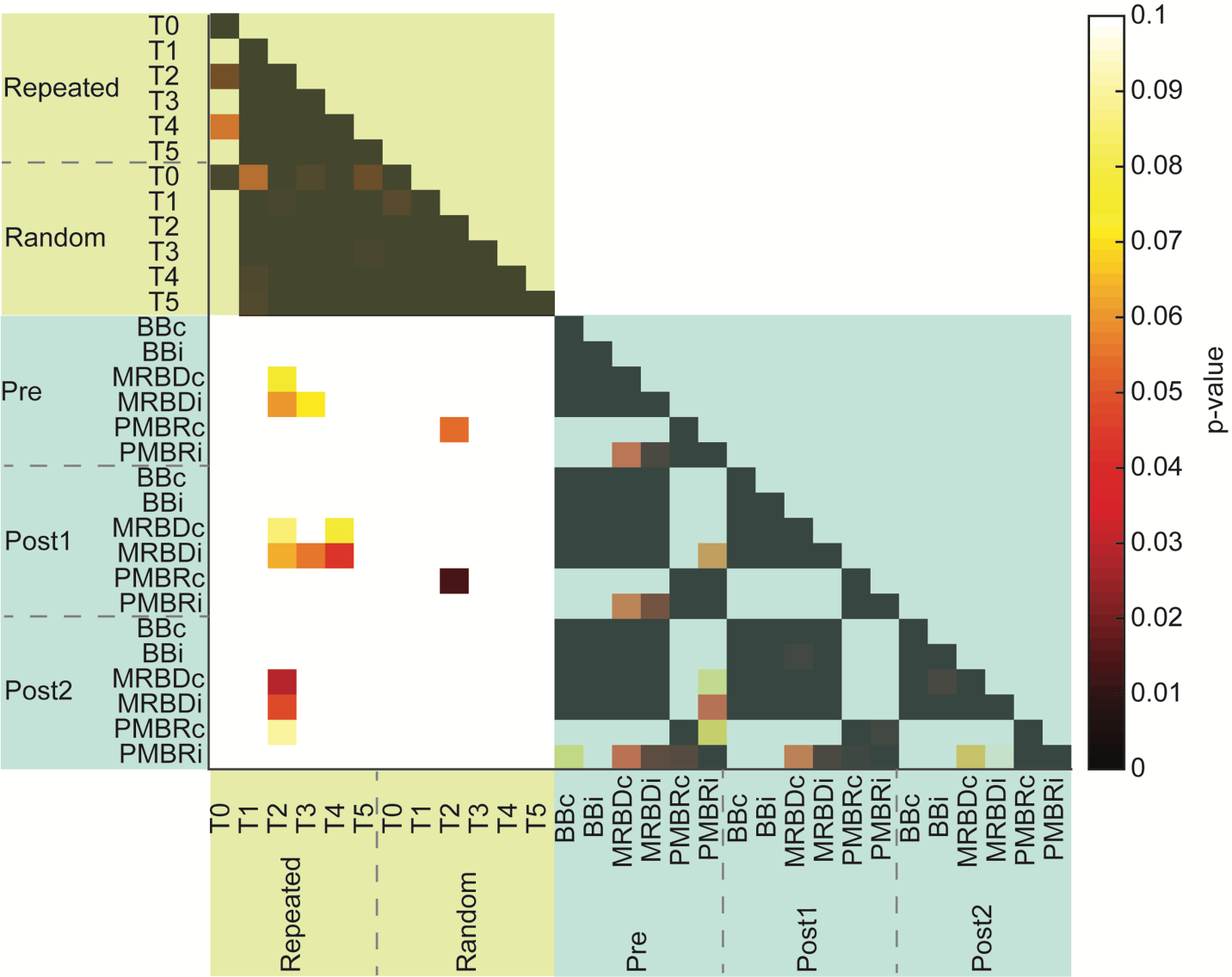


**Supplementary Figure 1:** Pairwise, uncorrected correlations between variables. P-values matrix of all correlations between neurophysiological measures (light blue box), motor performance measures (light green box), and between neurophysiological and motor performance measures. The background shading is based on the p-value of the associations (p<0.1), with darker colours representing smaller p-values. As expected, neurophysiological and motor performance measure are highly correlated. BBc and BBi: Pre-movement baseline beta from contralateral and ipsilateral sensorimotor cortex; MRBDc and MRBDi: Movement-Related Beta Desynchronization from contralateral and ipsilateral sensorimotor cortex; PMBRc and PMBRi: Post-Movement Beta Rebound from contralateral and ipsilateral sensorimotor cortex.


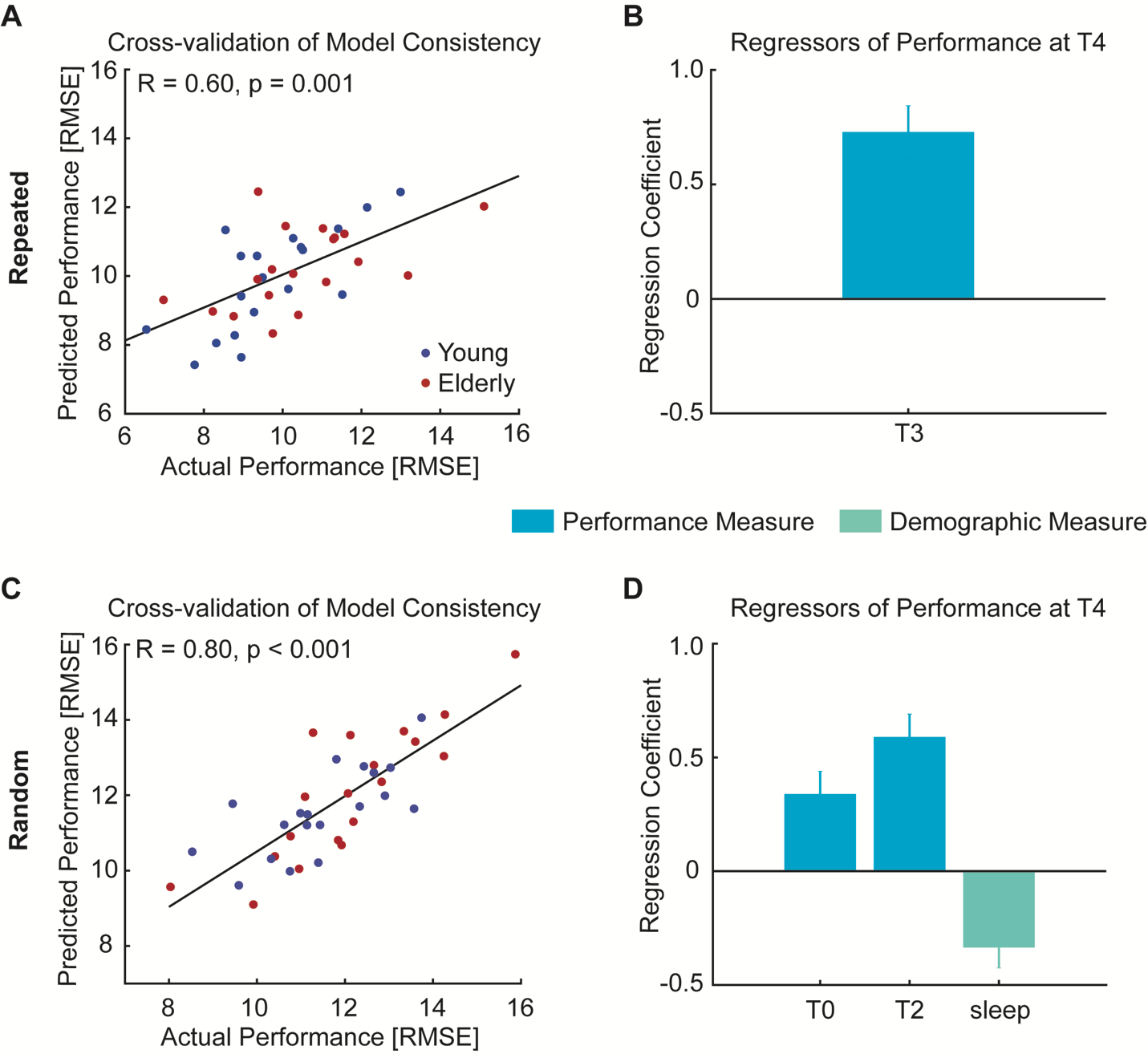


**Supplementary Figure 2:** Prediction of motor performance at T4. Stepwise multiple linear regression provided statistically significant performance prediction (**A, C**) as quantified by the correlation coefficient between the actual and predicted motor performance across healthy subjects. While solely motor performance accounted for 36 % of variance in performance on the repeated sequence at T4, a combination of motor performance and sleep measures explained 64 % of variance in performance on the random sequence. Significance of these correlations was determined by permutation-testing. **B**, Subjects’ performance at the end of retest1 alone significantly explained performance on the repeated sequence. **D**, Similarly, performance on the random sequence was strongly affected by model parameters relating to motor performance during training and retest. Interestingly, sleep quantity was associated with better performance on the random sequence at T4. Z-scored regression coefficients (β) quantify the influence of each significant predictor upon performance level at T2. Error bars represent SEM.
